# Experimental assessment of chemical solvents for asphaltic sludge removal to mitigate formation damage in oil reservoirs

**DOI:** 10.1038/s41598-025-06062-1

**Published:** 2025-07-01

**Authors:** Mojtaba Javdani, Sina Shakouri, Maysam Mohammadzadeh-Shirazi

**Affiliations:** 1 Department of Petroleum Engineering, School of Chemical and Petroleum Engineering, Shiraz University, Shiraz, Iran; 2 Formation Damage and Well Treatment Research Group, IOR/EOR Research Institute, Shiraz University, Shiraz, Iran

**Keywords:** Asphaltic sludge, Formation damage, Solubility, Tetrahydrofuran, Asphaltene

## Abstract

The reaction between acid and crude oil during oil well acidizing results in the formation of asphaltic sludge, a protonated complex enriched with H⁺ ions that forms within oil reservoirs. Its intricate structure and limited solubility make removal challenging, distinguishing it from asphaltene precipitation. This study aims to address the research gap regarding the effectiveness of solvents in asphaltic sludge removal. Three solvents, namely xylene, tetrahydrofuran (THF), and a kerosene-diesel mixture, were tested to evaluate their dissolution efficiency compared with the solubility of different asphaltene types. The results indicated that THF and xylene were the most effective solvents, with solubility rates of 70% and 60%, respectively. In contrast, the kerosene-diesel mixture exhibited nearly 0% solubility due to the absence of aliphatic and nonpolar compounds in the sludge required for effective dissolution. Analysis of influencing parameters revealed that increasing solvent volume to 20 mL/g improved solubility by 20%. In reservoir conditions, rock particle deposition is likely. The presence of 0.5 g of rock powders reduced THF and xylene solubility to 30% and 39%, respectively, due to H⁺ ion deactivation. Increasing contact time to 60 min and mixing speed to 1800 RPM further improved solubility by up to 10% and 20%, respectively.

## Introduction

One of the methods for permeability recovery in the near-wellbore area and stimulation of oil wells is acidizing. Acidizing refers to a process in which, by using fluid and the reaction of fluid with rock, dissolution of reservoir rock or near-wellbore areas occurs, and as a result, permeability is recovered^1^. Although matrix acidizing is an effective technique for permeability recovery and formation damage removal, it can cause new problems. The most important problems resulting from the matrix acidizing technique include equipment corrosion, precipitation, especially in sour reservoirs, instability, and asphaltene precipitation due to the dissolution of resins during reaction with hydronium ion and production of carbon dioxide gas, acid-oil emulsion, and asphaltic sludge, organic scaling caused by the incompatibility of pre-flush and post-flush fluids, wettability alteration, fine particle migration^2^. The formation of acid-oil emulsion and asphaltic sludge is recognized as the most prominent formation damage in oil wells and is among the most important reasons for the failure of acidizing operations^3^. In this process, when the injected acid comes into contact with reservoir oil, asphaltic sludge, or emulsion forms acid-induced damage^4^. To evaluate the formation of asphaltic sludge and estimate the extent of damage, a modified API RP-42 method has been used^5^.

Asphaltic sludge is a thick emulsion in which asphaltene-rich organic materials have been stabilized and is a mixture of various compounds that forms rapidly during the mixing of acid and oil, along with solid particles. Asphaltic sludge generally has a low molecular weight, is very small in size (less than 100 microns), and is not considered a polymeric material^6^. Petrographic images reveal that asphaltic sludge is a composite of oil, acid, iron ions, resin, and asphaltene, as well as saturated and aromatic compounds. Water is not present in this structure^7^. The results from gas pyrolysis chromatography indicate that saturated paraffins and unsaturated olefins with carbon numbers up to 30 are present in the structure of acid sludge^8^. The reaction rate for sludge formation in the process of producing sludge is very rapid, with the majority of it forming within the first 2 h. The phase transfer of acid into oil is initially very fast and then decreases after about 1 to 2 h. Experiments show that the reaction rate of sludge formation is faster than the phase transfer rate^7^. The properties of asphaltic sludge and asphaltene are described in Table 1 in order to demonstrate the main components and differences of these two compounds^9,10^.

**Table 1:** Physical and chemical characteristics of asphaltic sludge and asphaltene.

Characteristic	Asphaltic sludge	Asphaltene
Physical Appearance	Sticky, shiny, viscous emulsions can also appear as bulk sediment	Dark brown to black, brittle, solid in dry form; forms stable colloids in crude oil
Composition	Enriched with asphaltenes, resins, aromatics, and saturates, polar groups (C–O, C–S, C–N) dominate	Composed mainly of polyaromatic hydrocarbons with heteroatoms (N, S, O); contains metals like vanadium and nickel
Particle Size	Particles > 300 nm precipitate; smaller particles remain stable	Aggregates typically range from 2 to 100 nm in crude oil; they can form larger flocs under instability
Viscosity	Potentially immobilizing	Exhibits high viscosity when dispersed in crude, contributing to flow resistance and blockages
Wettability Alteration	Alter the rock wettability from water-wet to oil-wet	Strong tendency to alter rock wettability toward oil-wet in porous media
FTIR Analysis	FTIR spectra reveal stronger absorption bands in the 1000–1200 cm^−1^ range for sludge compared to the parent crude oil, indicating higher contributions of polar functional groups	FTIR shows absorption peaks associated with aromatic C=C stretching (~ 1600 cm^−1^) and polar groups (C = O, N–H)
Iron Contamination	Ferric (Fe^3^⁺) and ferrous (Fe^2^⁺) ions strongly enhance sludge formation, even at low concentrations	None

Research indicates that various parameters of acid and oil affect the process of asphaltic sludge formation. These parameters include the type and strength of the acid, Type of iron ion and its concentration, Temperature, Duration of acid-oil contact, Oil density and viscosity, Surface tension between acid and oil^3,11^. Other studies indicate that multiple factors play a role in this complex process. The physicochemical properties of crude oil, including viscosity, H_2_S gas content, and SARA (Saturate, Aromatic, Resin, Asphaltene) component composition, along with operational parameters such as pressure, temperature, and acid type, directly influence sludge formation^12,13^. The molecular structure of oil, including the polarity and spatial arrangement of components, has been recognized as one of the main controlling factors in the formation of asphaltic sludge^14,15^. Based on the research conducted, the presence of asphaltene and the interaction between asphaltene and resin have a direct impact on the formation of asphaltic sludge^16^. Acid concentration, presence of iron ions, and acid-to-mixture ratio have been reported as the main factors affecting emulsion stability and sludge amount^17^. In a study conducted, the effect of paraffinic and aromatic components of crude oil on the production of acid sludge was investigated. To examine this effect, synthetic oil was used to isolate the impact of crude oil components. In the continuation of this study process, solvents such as DMF, acetone, tetrahydrofuran, dichloromethane, and toluene were used to investigate the effects of their components on acid sludge production^18^.

**Fig. 1:**
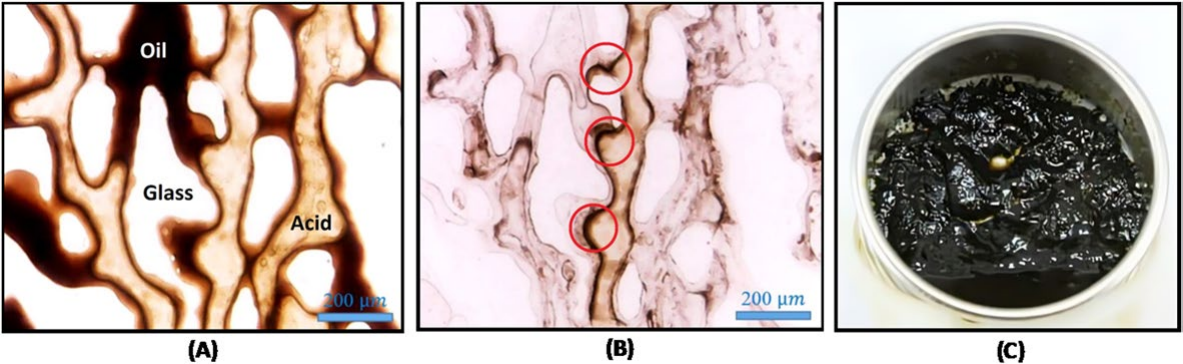
Glass micromodel saturated with crude oil following injection of 15 wt.% HCl. (**a**) shows the system after 2 h of retention time, and (**b**) shows it after flushing with water and diesel. Dark brown regions (highlighted with a red circle) indicate asphaltic sludge deposition at pore walls^22^. (**c**) shows asphaltic sludge formed on a metal mesh.

In a study, it was examined that improper design of acidizing operations leads to the formation of stable acid-oil emulsions and asphaltic sludge. This study focused on the formation of emulsions and their stability, as well as the factors affecting the formation of asphaltic sludge, such as acid injection rate to the reservoir, acid volume percentage, temperature, and oil viscosity. The results indicated a direct relationship between mixing speed and temperature with emulsion stability, while an inverse relationship was found between AMR (Acid Volume Ratio) and emulsion stability^10^. In a study by Abbasi, et al.^19^, the stability of acid-oil emulsions was investigated under the influence of the pH of the injected acid. The results of this research indicated that the pH of the injected HCl solution increases during its reaction with rock and mineral materials. In this context, Abbasi, et al.^20^ examined the impact of several chemical additives, including corrosion inhibitors, corrosion inhibitor intensifiers, and iron control agents, on the damage caused by asphaltic sludge formation. The results indicated that although the additives are effective for their primary functions, they delay emulsion phase separation by 30 min. Acid stimulation, as one of the methods to enhance recovery from oil reservoirs, leads to the formation of stable acid-oil emulsions. This study investigates the effect of the presence of asphaltene on the stability of emulsions. According to reported research, a small portion of asphaltene, known as interfacial-active asphaltene (IAA), is the primary stabilizing agent for water–oil emulsions. The results obtained indicate that the lower the amount of interfacial-active asphaltene (IAA), the lower the stability of the emulsion will be^21^. Figure 1 shows the formation and deposition of asphaltic sludge on pore-scale surfaces and metal mesh following acid treatment, providing visual confirmation of sludge accumulation in porous media representative of oil reservoir conditions.

The formation of asphaltic sludge has destructive and significant effects on oil wells, causing numerous problems, such as increased acid injection pressure required for acid to enter the formation, observation of thick and high-viscosity materials in return pipes, unintended creation of new pathways behind the casing, and unsuccessful acidizing and well stimulation operations^8,17^. During the stability of hard acid-oil emulsions and the subsequent formation of asphaltic sludge, permeability decreases, and the viscosity of the emulsion increases. In this context, the wettability of the rock also changes, resulting in a reduction in relative permeability.

In the oil industry, thermal methods and solvents are used to remove organic deposits in oil wells. Among these, using solvents is a cost-effective and efficient method for oil wells. The term solvent refers to a substance that is capable of dissolving or solubilizing another substance within itself^23^. While there have been limited studies specifically on the solubility of asphaltic sludge, numerous research efforts have focused on the solubility of asphaltene deposits. To date, various types of chemical solvents have been used in the industry to remove asphaltene deposits. The most important and cost-effective ones include Xylene, Toluene, Kerosene, Diesel, Terpene, Tetrahydrofuran, and Esters^24,25^. In the first laboratory research, an experiment was designed to investigate the solubility of asphaltene. This experiment aimed to find the best type of solvent for removing asphaltene deposits. The experiment includes an equation that outputs the percentage of asphaltene solubility with the solvent in question^24^. In another study, a binary mixture of n-heptane was used to investigate the removal of asphaltene deposits. This mixture included chemical compounds such as benzonitrile, cyclohexanone, ethyl benzoate, 1-methyl naphthalene, tetrahydropyran, and toluene. As a result, efficient additives that do not alter the viscosity and stability of the water–oil emulsion must be utilized^26^. One of the solvents investigated for removing asphaltene deposits in porous media is a toluene-water emulsified solvent. To study this type of solvent, three different emulsions were prepared with water-in-oil volume percentages of 90/10, 80/20, and 70/30. These emulsions were injected into a transparent heterogeneous micromodel containing asphaltene deposits at injection rates of 0.1 and 1.0 cc/hour. The result of these experiments led to the removal of a significant amount of asphaltene deposits, and the 70/30 water-in-oil emulsion was selected as the optimal solvent^27^. In the latest studies, a novel method called TPMDS (Toluene-based Polar Microemulsion Dispersant System) was introduced for removing asphaltene deposits, which can be implemented in both static and dynamic conditions. This method includes solvents such as toluene, methanol, surfactant, dodecyl-benzene sulfonic acid, and sodium hydroxide. In this experiment, static conditions proved to be the most effective for asphaltene deposit removal. The effectiveness of this combined package of various chemical solvents increases the solubility and removal of deposits to 84 percent, and the rock permeability increases by 290 percent^28^. In another study, solubility tests were conducted on a specific asphaltene sample using heptane, pentane, and a mixture of these with toluene, which resulted in varying effectiveness in eliminating asphaltene deposits^29^. Moreover, recent studies have demonstrated that nonionic surfactants can aid in the breakdown of asphaltene agglomerates through peptization and interfacial mechanisms^30,31^. This supports the relevance of comparing surfactant effects in similar systems.

To the best of the authors’ knowledge, there has been no prior research on chemical solvents for the removal of asphaltic sludge deposits. However, a case study in 1999 mentioned THF as a solvent for asphaltic sludge, but it did not provide any precise, reliable, or noteworthy results. Additionally, no comparison was made with other solvents; THF was merely referenced as a solvent without further details. In this research, solvents for the removal of asphaltic sludge have been proposed based on studies conducted on asphaltenes. Various experimental designs centered on solubility tests were employed to identify the best solvent and to examine different types of solvents for eliminating asphaltic sludge. The study also investigated the impact of various parameters and factors on solubility, such as temperature, mixing speed, the volume ratio of solvent to solute, the presence of rock powder, the type of solvent, the nature of asphaltic sludge, and the duration of contact between the solution and asphaltic sludge. Laboratory results indicated that tetrahydrofuran (THF) and xylene are effective solvents for asphaltic sludge deposits. The solubility was found to have a direct relationship with the duration of contact between the solution and the sludge, the volume ratio of solvent to solute, and mixing speed, while it showed an inverse relationship with the presence of rock powder.

## Material and methods

### Materials

#### Asphaltic sludge and asphaltene

In this research, three oil samples from an oil field in the southwest of Iran, where asphaltic sludge formation is possible, were utilized. Following this, asphaltic sludge and asphaltenes were extracted under various conditions from each of the oil samples during additional experiments. The characteristics of crude oil samples are shown in Table 2. The conditions and properties of asphaltic sludges formed under various circumstances are detailed in Table 3. Also, in Table 4, the properties of asphaltene extracted from the collected crude oil samples are presented. Among these, the AMR parameter is a key factor in the conditions for asphaltic sludge formation, representing the acid-to-oil ratio in the experiments.

**Table 2:** The properties of the crude oil samples used in this study.

Property	Crude oil A	Crude oil B	Crude oil C
API gravity	20.3	27.86	30.06
Saturation	47.74	47.24	45.69
Aromatic	37.19	38.19	40.88
Resin	6.52	7.03	8.03
Asphaltene	8.55	7.54	5.41
Viscosity (cp) @ 25 °C	140	56	17
Viscosity (cp) @ 60 °C	23	2.3	1.5

**Table 3:** The properties of the asphaltic sludge used in this study.

Sludge	Acid mixture ratio	Mixing speed (rpm)	Temperature (°C)	From Sample
S1	0.5	1500	85	Crude oil A
S2	0.5	500	85	Crude oil A
S3	0.5	1000	85	Crude oil A
S4	0.5	1500	70	Crude oil A
S5	0.5	1500	30	Crude oil A
S6	0.5	1500	50	Crude oil B
S7	0.2–0.5–0.8	1500	85	Crude oil B
S8	0.5	1500	50	Crude oil C

**Table 4:** The properties of extracted Asphaltenes.

Sample no	Source crude oil	Extraction method (standard)	Color/form	Onset precipitation temp (°C)	Solubility (n-heptane/toluene)
A1	Crude oil A	ASTM D6560/API 141	Black, Powder	~ 60	Insoluble in n-C7, Soluble in Toluene
A2	Crude Oil B	ASTM D6560/API 141	Brown-Black, Granular	~ 52	Partial insolubility in n-C7
A3	Crude Oil C	ASTM D6560/API 141	Dark Brown, Fine Dust	~ 65	Fully Soluble in Toluene

#### Solvents

In this research, various solvents were employed, as detailed below. Additionally, the properties of these solvents are described in Table 5. It should be noted that a 50–50 kerogen-diesel solvent mixture was utilized in the experiments.

1. *Xylene*: A clear, water-insoluble liquid with irritating vapors. It belongs to the group of aromatic compounds and is used as a chemical solvent.
2. *Tetrahydrofuran*: An organic compound that is colorless and miscible with water, used as a polar organic solvent.
3. *Kerosene*: A petroleum-based compound and fuel, it is a flammable liquid hydrocarbon.
4. *Diesel*: The liquid fuel used in diesel engines, obtained through the fractional distillation of heavy crude oil.

**Table 5:** The solvents used in this study and their properties.

Chemical group	Name	Polarity	Purity (%)	Boiling point (°C)	Supplier
Aromatic	Xylene	2.5	99	137–143	Dr. Mojallali Industrial Chemical Complex Co
Non-aromatic	THF	4	99	66	
Aromatic	Kerosene	Non-polar	–	150–275	
Aromatic	Diesel fuel	Non-polar	–	180–360	

### Methods

#### Extraction of asphaltenes

For the production and extraction of asphaltenes from any oil sample, API Standard 141 is utilized:

1. For every one cc of crude oil, 40 times the volume of normal heptane is added, and it is stirred on a magnetic stirrer for 12 h.
2. After mixing is complete, the container is covered with aluminum foil and placed in a dark location for 24 h.
3. Steps 1 and 2 are repeated a total of three times.
4. The mixture of crude oil and normal heptane is then filtered using Whatman No. 40 filter paper and a vacuum pump.
5. The filter paper containing the residues is placed in a Soxhlet extractor, and the deposits on the surface of the filter paper are first washed with normal heptane and then with toluene. Each washing step continues until the color of the fluid in the siphon becomes clear.
6. Finally, the toluene containing asphaltenes is poured into a petri dish and placed under a fume hood to allow the toluene to evaporate, resulting in the collection of pure asphaltenes.

#### Solubility test

Solubility tests are employed to investigate the effect of solvents on the removal of asphaltic sludge deposits. The objective is to examine the solubility power of asphaltic sludge in the mentioned solvents, considering both static and dynamic solubility conditions.

- *Static Condition*: This refers to determining the solubility of asphaltic sludge in the solvent without stirring the solution.
- *Dynamic Condition*: This involves stirring the solution at a specified speed.

Due to the impact of the contact time of asphaltic sludge with the solvent on solubility, experiments are conducted dynamically for two-time intervals: 30 min and 1 h. According to preliminary tests, under these conditions, surface evaporation is negligible and can be disregarded. Additionally, because temperature affects the solubility of asphaltic sludge, the solution is maintained at a temperature of 40 °C, which is then increased to 90 °C for drying the filter paper. In this experiment, solvents such as toluene, kerosene-diesel, and tetrahydrofuran are utilized^6^.

The method for conducting the solubility test is as follows:

1. 0.05 g of asphaltic sludge is placed in contact with 5 ml of the specified solvent.
2. The solution, along with a magnet, is placed on a stirrer for 3 min at speed 4.
3. The solution is placed in an oven until the temperature reaches 40 degrees Celsius (it should remain in the oven for 30 min).
4. The filter paper is placed in the oven at 90 degrees Celsius for about 1 h and weighed three times until its weight stabilizes (initial weight). Whatman No. 40 filter paper was used in the experiments.
5. The solution is poured onto the filter paper.
6. The filter paper is placed in the oven at 90 degrees Celsius and weighed three times until its weight stabilizes (secondary weight).
7. Finally, to calculate the percentage of solubility of the asphaltic sludge, the following formula is used:
  $${\text{Solubility}}\,{\text{of}}\,{\text{Asphaltic}}\,{\text{Sludge}}\% = 100 - \left( {\frac{{{\text{Secondary}}\,{\text{Weight}} - {\text{Initial}}\,{\text{Weight}}}}{{{\text{Amount}}\,{\text{of}}\,{\text{Solvent}}}}*100} \right)$$

## Results and discussion

Initially, it is important to understand that asphaltic sludge is not the same as asphaltene and should not be confused with it. Asphaltic sludge is a more aromatic substance that is formed as a result of the mixing of acid with oil and is a heavier compound than asphaltene, even though asphaltene is also present in this mixture.

In this section, the experimental results regarding the solubility of various samples of asphaltic sludge and asphaltene are presented in detail. The solubility of asphaltic sludge and asphaltene is shown in two different tables, and these results are compared in various graphs. Additionally, the effects of stirring speed, the presence of stone powder, solvent volume, and the duration of solvent contact are examined, and the results are provided. During these experiments, three different types of asphaltene were used: samples A1 and A2 correspond to asphaltene extracted from two oil wells, while sample A3 is an industrial sample of asphaltene.

The asphaltic sludge samples tested in this study are derived from the asphaltic sludge of three oil wells in the southwest of Iran. Eight different asphaltic sludge samples extracted under various conditions were used in this study. It should also be noted that asphaltic sludge sample number 7 was not used in the experiments, as the desired results had already been achieved in previous tests. However, due to its overlap with previous research in the field of asphaltic sludge extraction, it has been included here (Table 3)^10^. The properties of the asphaltic sludge are presented in Table 1. The only difference between the asphaltic sludge samples is the method of extraction under different conditions.

### Solubility of asphaltic sludge

In this section, the solubility results of asphaltic sludge using various solvents are presented and discussed. The solubility of each sample was measured using different solvents according to the solubility test described in the previous section. In this context, several experiments were designed and conducted to measure the solubility of asphaltic sludge, during which parameters such as the solvent-to-solution ratio, solvent and solution contact time, and temperature were also evaluated.

As shown in Tables 6 and 7, the percentage solubility for each test is provided based on the type of solvent and the constancy of other parameters such as the solvent-solution ratio, solvent and solution contact time, and temperature. It should be noted that all experiments were conducted dynamically at a constant temperature of 40 °C with a contact time of 30 min. In this context, several preliminary experiments were conducted to determine the optimal conditions for each parameter, as presented in Table 6.

**Table 6:** Results of preliminary Tests.

Test	Sample	Solvent	Conc	Sludge/solvent (g/ml)	Test type	Temp	Solubility %
1	S1	Xylene	100%	0.05/10	Dynamic	70	87%
2	S1	Xylene	100%	0.0281/5.6	Dynamic	85	91%
3	S2	THF	100%	0.05/10	Static	30	99.35%
4	S2	Kerosene + Diesel fuel	50% + 50%	0.05/10	Static	30	92.34%

**Table 7:** The results of the effect of solvent type on the solubility of asphaltic sludge (Dynamic test, T = 40 °C).

Test	Sample	Solvent	Conc	Sludge/solvent	Solubility %
5	S3	Xylene	100%	0.05/10	66.2%
6	S3	Xylene	100%	0.05/10	57%
7	S5	Kerosene + Diesel fuel	50% + 50%	0.05/10	0%
8	S5	Kerosene + Diesel fuel	50% + 50%	0.05/10	0%
10	S5	THF	100%	0.05/10	65.5%
11	S5	THF	100%	0.05/10	77%
12	S4	THF	100%	0.05/10	49.2
13	S8	Xylene	100%	0.05/10	35.4
14	S6	Xylene	100%	0.05/10	59.8
15	S6	Xylene	100%	0.05/10	63.2
16	S6	Kerosene + Diesel fuel	100%	0.05/10	0
17	S6	THF	100%	0.05/10	68.2
18	S6	THF	100%	0.05/10	65.6
19	S6	Xylene + THF	50% + 50%	0.05/10	63.2

Based on the results presented in Table 7, regarding the solubility of asphaltic sludge, it can be inferred that among the solvents used, tetrahydrofuran exhibits greater solubility compared to xylene and the combination of kerosene and diesel. To investigate the reason for this, one can refer to its higher polarity relative to other solvents and its aromatic composition similarity to that of asphaltic sludge. As observed in Table 7, the solubility of tetrahydrofuran varies between 65.5 and 77%, while the solubility of xylene ranges from 57 to 66.2%, which differs according to the properties of asphaltic sludge. An important point is that combining two solvents does not significantly impact the results. Furthermore, the combination of kerosene and diesel, as a non-polar and alkane solvent, indicates that there is no solubility effect on asphaltic sludge, resulting in a very negligible solubility close to zero since asphaltic sludge is a completely polar compound. Additionally, combining tetrahydrofuran and xylene does not yield greater solubility in asphaltic sludge, even if the sample of asphaltic sludge changes.

**Fig. 2:**
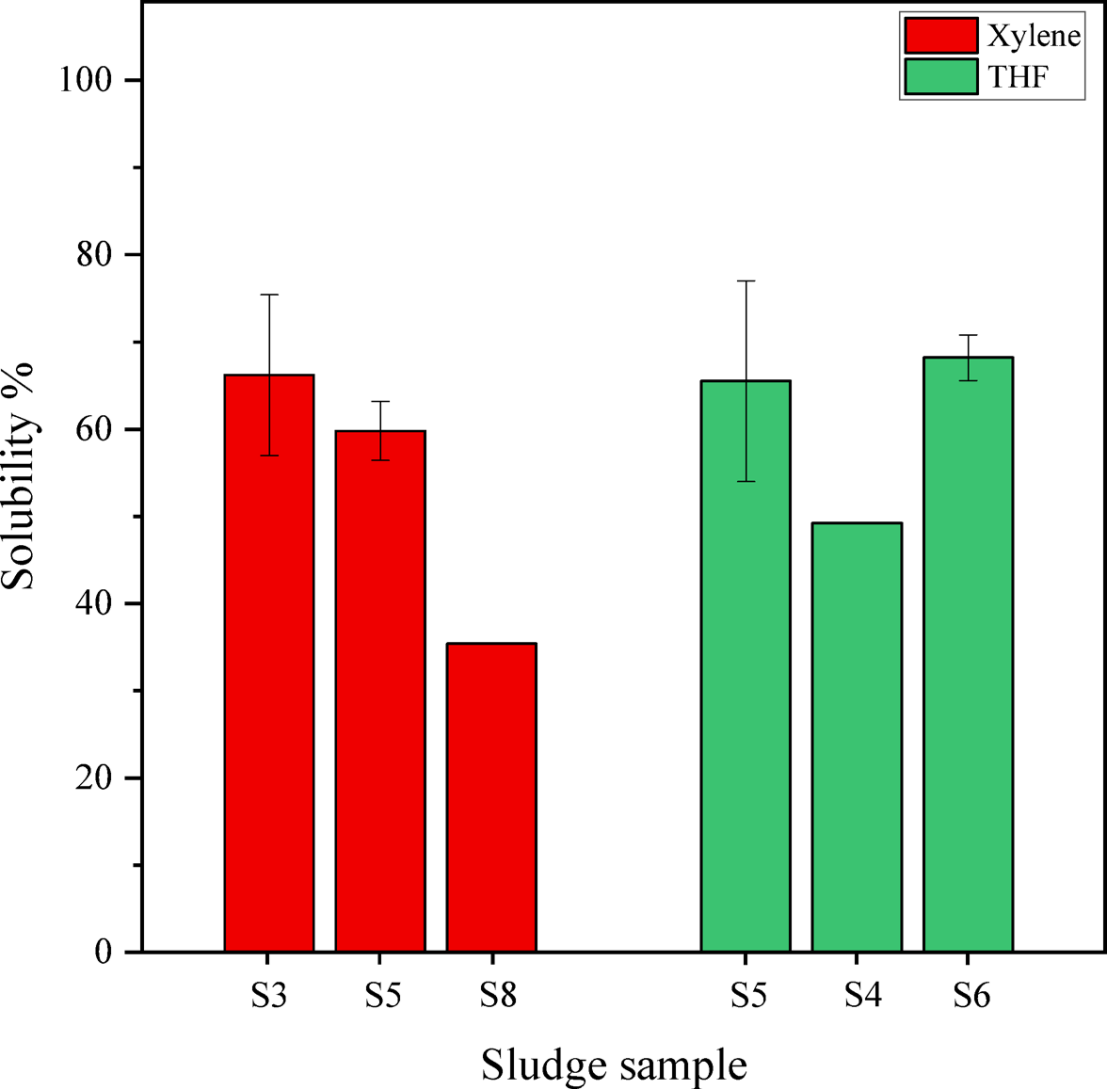
The solubility results of five samples of asphaltic sludge S3, S4, S5, S6, and S8 in the presence of two types of solvents: xylene and tetrahydrofuran (Dynamic test, T = 40 °C).

To provide a clearer examination of the effect of solvents on the solubility of asphaltic sludge, the following graph (Fig. 2) has been utilized.

As shown in the red section of the bar chart in Fig. 2, the solubility of xylene has been examined on three different types of asphaltic sludge. The solubility of xylene ranges from 57 to 66.2%, which varies depending on the type of asphaltic sludge and the ions present in it. The higher the amount of AMR and temperature during the formation of asphaltic sludge, the greater the solubility will be.

As shown in the green section of the bar chart in Fig. 2, the solubility of tetrahydrofuran has been examined on three types of asphaltic sludge. The solubility of tetrahydrofuran is slightly higher than that of xylene, indicating better performance compared to xylene, with a solubility range of 65.5–77%. This is because tetrahydrofuran is a more polar and aromatic compound, which results in greater solubility.

### Solubility of asphaltene

In this section, the solubility results of asphaltene are presented and discussed using various solvents. All necessary conditions and parameters for conducting these tests, such as the solubility testing conditions for asphaltic sludge, have been considered to allow for comparison in the analysis of the solubility of asphaltic sludge and asphaltene. The solubility of each sample was measured using different solvents according to the solubility test described in the previous section. In this part, solubility tests were conducted on two laboratory-extracted asphaltene samples and one industrial asphaltene sample, with the results fully presented in Table 8.

**Table 8:** Results of asphaltene solubility (Dynamic test, T = 40 °C).

Test	Sample	Solvent	Asphaltene/solvent (g/ml)	Conc	Solubility %
1	A3	Xylene	0.05/10	100%	100%
2	A3	Xylene	0.05/10	100%	100%
3	A3	Kerosene + Diesel fuel	0.05/10	50% + 50%	6.2%
4	A3	Kerosene + Diesel fuel	0.05/10	50% + 50%	0%
5	A3	THF	0.05/10	100%	100%
6	A3	THF	0.05/10	100%	100%
7	A3	Xylene	0.05/10	100%	69%
8	A3	THF	0.05/10	100%	82.8%
10	A3	Kerosene + Diesel fuel	0.05/10	50% + 50%	0%
11	A1	Xylene	0.05/10	100%	94.4
12	A1	Xylene	0.05/10	100%	92.4
13	A1	THF	0.05/10	100%	79.2
14	A1	THF	0.05/10	100%	72
15	A2	Xylene	0.05/10	100%	89.8
16	A2	Xylene	0.05/10	100%	94.6
17	A2	THF	0.05/10	100%	82.2
18	A2	THF	0.05/10	100%	86.4

So far, various articles have been published in the field of asphaltene solubility. In this section, the obtained results are similar to previous findings in this area, confirming the validity of the experiments. Generally, different solvents are used to eliminate asphaltene deposits. Elochukwu and Mahmud^32^ conducted a study to find an alternative solvent with lower biodegradability issues and reduced toxicity. As a result of this study, a mixture of methyl ester oleate and ethanol was introduced as a substitute solvent. This solvent is environmentally friendly, has a high flash point, and demonstrates excellent solubility for asphaltene deposits. In another study, the use of emulsified solvents (toluene emulsions in water) was investigated as an alternative approach for removing asphaltene deposits in porous media. In this study, a comparison was made between emulsified solvents and pure toluene, and it was found that the emulsified solvent dissolved more asphaltenes due to its ability to penetrate deeper^27^.

As shown in Table 8, the best solvents for removing asphaltene deposits are xylene and tetrahydrofuran. These two solvents exhibit higher solubility due to their high polarity and reactivity. The solubility results for xylene indicate that the solubility is 95%, and sometimes this solubility can reach 100% depending on the type of asphaltene and its properties. The solubility results for tetrahydrofuran show that the average solubility is around 80%, which can be considered as a substitute for xylene^33^. Another solvent that has been used to investigate asphaltene solubility is kerosene and diesel. The combination of these two solvents has a very minimal effect on the solubility of asphaltene deposits, with the solubility of this mixture being less than 5%.

**Fig. 3:**
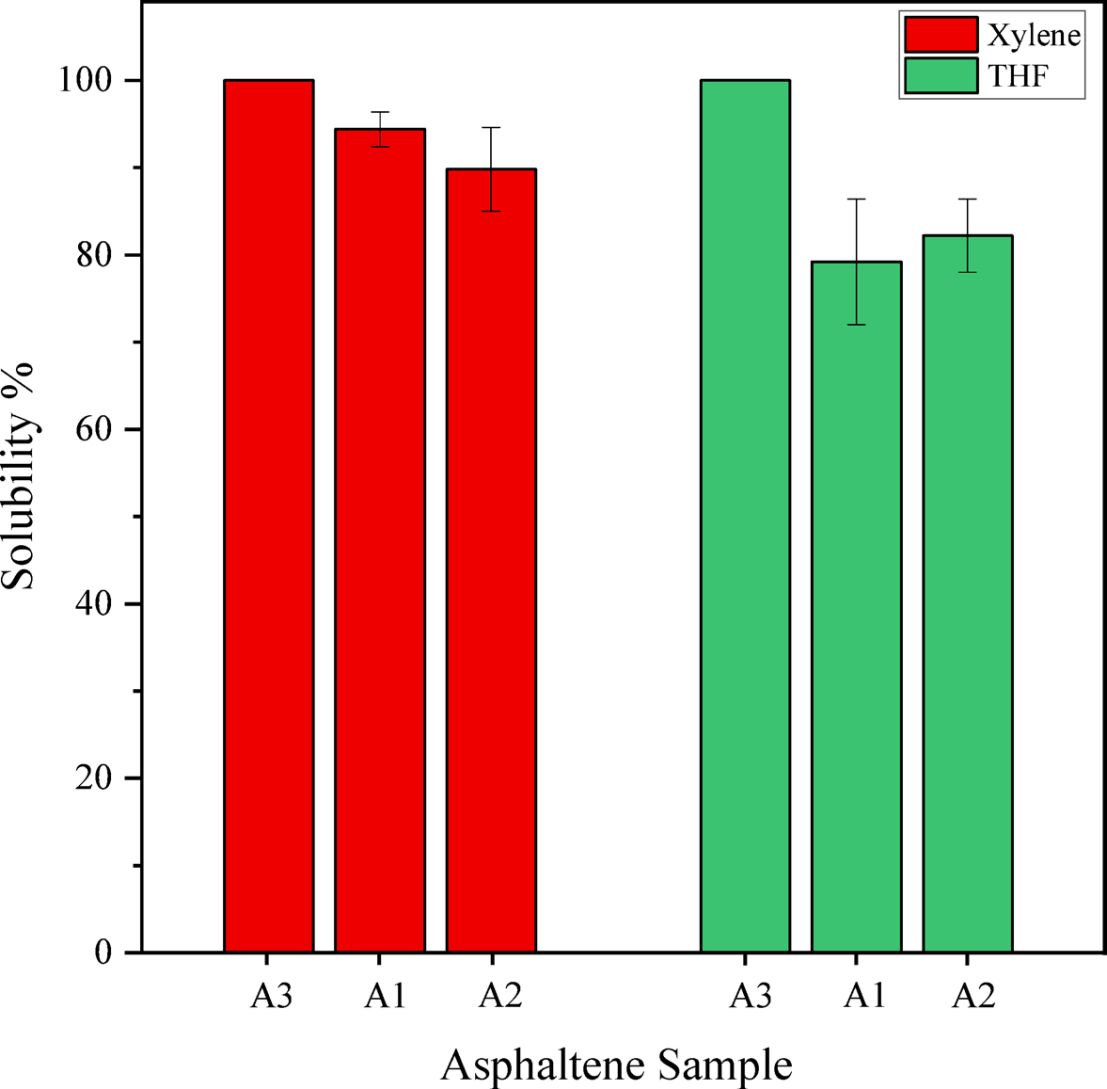
Results of the solubility of three types of asphaltene A1, A2, and A3 in the presence of two types of solvents: xylene and tetrahydrofuran (Dynamic test, T = 40 °C).

To provide a clearer examination of asphaltene solubility, the following graph (Fig. 3) has been utilized.

As shown in the red section of the bar chart in Fig. 3, the solubility of xylene has been examined on all three types of asphaltenes. According to the obtained results, which confirm previous articles and experiments conducted by others, xylene demonstrates very good solubility for asphaltene, with an average solubility percentage of 95%^32^.

In the green section of the bar chart in Fig. 3, as observed, the solubility of tetrahydrofuran has been examined on all three types of asphaltenes. According to the obtained results, tetrahydrofuran performs similarly to xylene and shows good solubility; however, due to its lower boiling point and higher cost, it is rarely used in industry for removing asphaltene deposits, with an average solubility of 80%.

### Effect of the presence of stone powder, the speed of the stirrer, the duration of exposure, and the amount of solvent

To investigate the effect of various parameters on solubility, a series of experiments was designed, all conducted in the same manner as before. The effects of different parameters, such as the presence of powdered stone, stirring speed, duration of contact, and the amount of solvent, were tested as follows:

- *Stirring Speed*: To assess the impact of stirring speed (reaction rate) in dynamic testing, experiments were conducted using a stirrer at two speeds: 400 and 1800 RPM.
- *Amount of Solvent*: The influence of solvent quantity on solubility was tested at different volumes of 5, 10, and 20 cc.
- *Duration of Contact*: For this parameter, the solvent and solution were placed together at a temperature of 40 degrees Celsius for durations of 30 and 60 min.
- *Presence of Powdered Stone*: To evaluate the effect of foreign substances and the presence of new ions on the reaction rate, powdered stone was used, and its results were analyzed.

As shown in Table 9, the overall results of the experiments regarding the impact of the presence of powdered stone, stirring speed, duration of contact, and the amount of solvent are presented. To examine the effect of the presence of powdered stone, half the weight of the asphaltic sludge was added to the powdered stone solution. For investigating the effect of duration of contact, the contact time was increased to 60 min, and the results were recorded. In subsequent experiments, to evaluate the stirring speed, this parameter was increased to 1800 RPM. Finally, to assess the effect of solvent quantity, the amount of solvent was changed to 5 and 20 cc. It should also be noted that asphaltic sludge samples numbers 4 and 5 were combined due to the consistency of their properties^10^.

**Table 9:** Results of solubility tests in evaluating various parameters (Dynamic test, T = 40 °C).

Test	Sample	Solvent	Solvent volume (cc)	Rock flour (g)	Solving time (min)	Mixer RPM	Solubility %
1	S6	Xylene	10/0.05	0.5	30	400	38.5
2	S6	Xylene	10/0.05	0.5	30	400	31.8
3	S6	THF	10/0.05	0.5	30	400	23.8
4	S6	THF	10/0.05	0.5	30	400	28.9
5	A2	Xylene	10/0.05	0	60	400	91.5
6	A2	Xylene	10/0.05	0	60	400	96.5
7	S6	Xylene	10/0.05	0	60	400	67.1
8	S6	Xylene	10/0.05	0	60	400	56.8
9	S4 + S5	Xylene	10/0.05	0	30	1800	45.7
10	S4 + S5	Xylene	10/0.05	0	30	1800	43
11	S6	Xylene	10/0.05	0	30	1800	71.3
12	S6	Xylene	10/0.05	0	30	1800	70.1
13	S6	Xylene	20/0.05	0	30	400	86
14	S6	Xylene	5/0.05	0	30	400	54.6

**Fig. 4:**
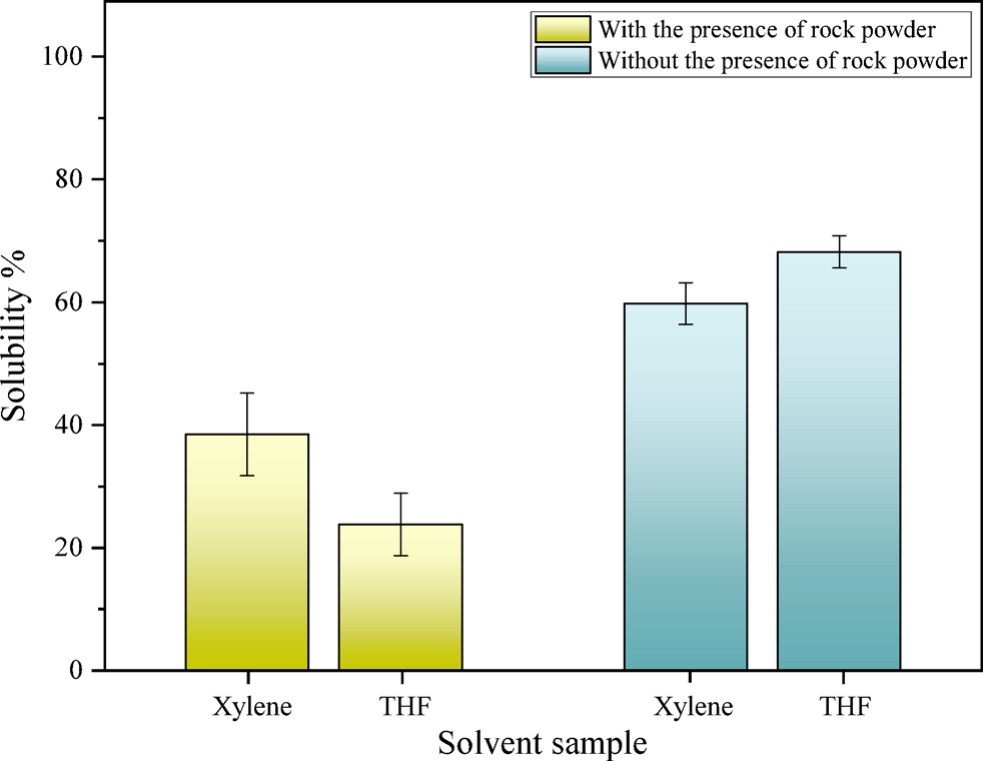
Results of the effect of the presence of powdered stone (Dynamic test, T = 40 °C).

To more clearly and accurately examine the effects of various parameters, graphs are also utilized. As shown in Fig. 4, the presence of powdered stone causes the ions present in tetrahydrofuran and xylene to be absorbed by the surface of the stone, effectively reducing their reactivity and ability to penetrate the asphaltic sludge. The reaction between tetrahydrofuran ions and powdered stone is greater than that with xylene, resulting in lower solubility for tetrahydrofuran compared to xylene. Overall, the presence of powdered stone reduces the solubility of tetrahydrofuran from 65.5 to 28.9%, while the solubility of xylene decreases from 57 to 38.5%. Consequently, solubility decreases in the presence of powdered stone.

**Fig. 5:**
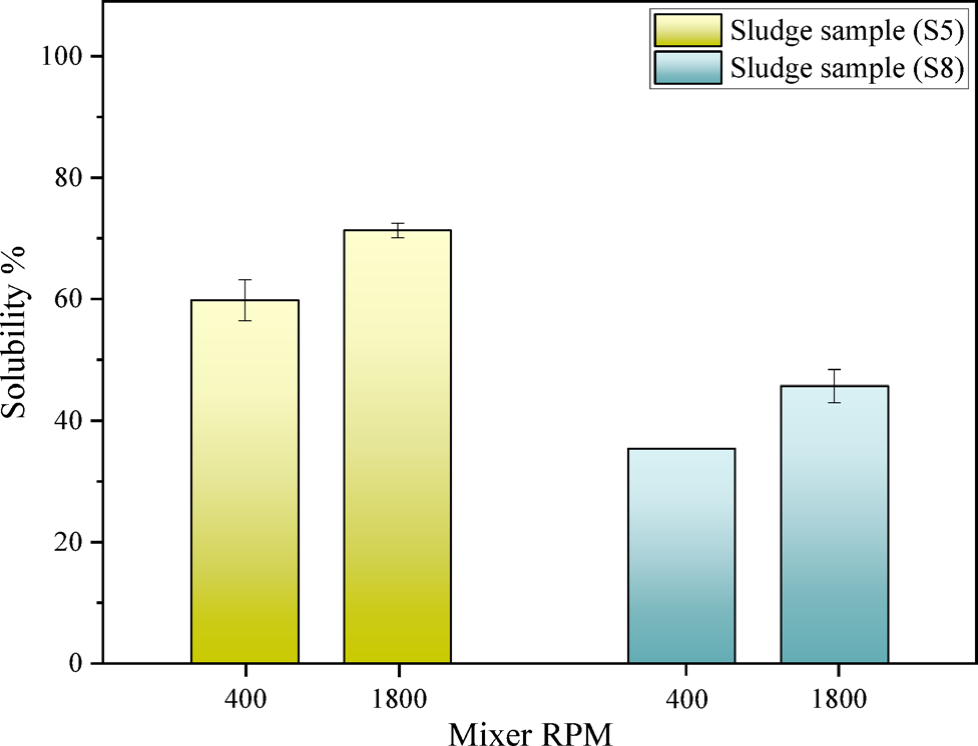
Results of the effect of stirring speed (Dynamic test, T = 40 °C).

As shown in Fig. 5, with an increase in stirring speed, molecular motion and the collision of molecules due to an external factor increase, resulting in a higher reaction rate. Over a specified period, more molecules of the solvent and solute react with each other, leading to an increase in solubility. This result is also confirmed by previous studies, which indicate that based on prior knowledge and experience, an increase in stirring speed enhances solubility. In both samples, the solubility of asphaltic sludge increased with the rise in stirring speed, resulting in a 15% increase in solubility. The exact difference between static and dynamic solubility tests^28,34^.

**Fig. 6:**
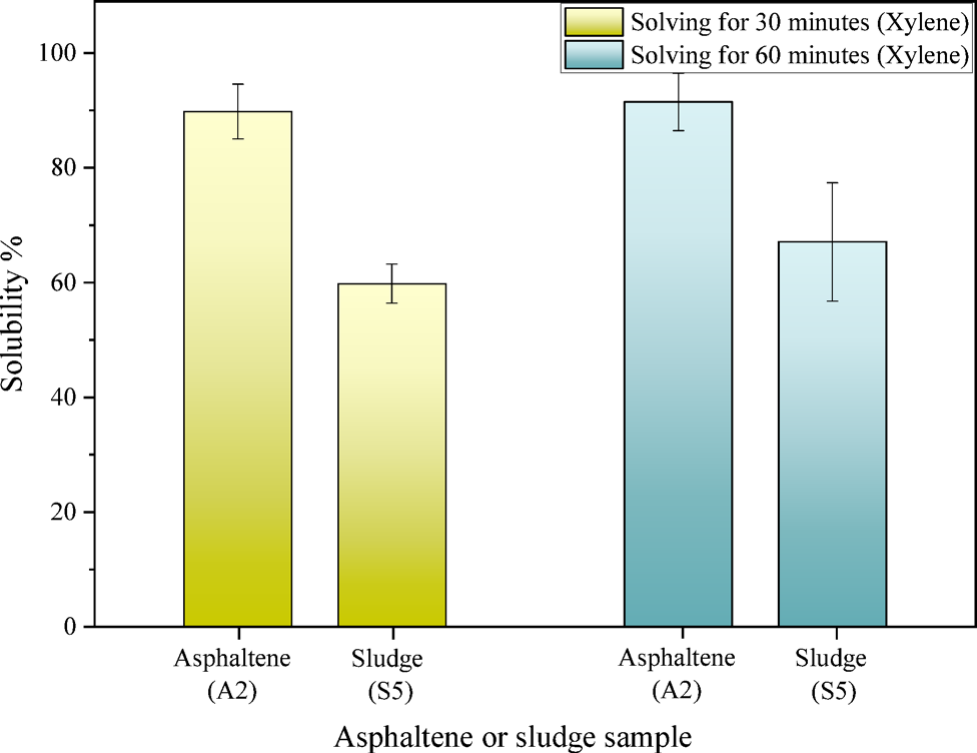
Results of the effect of duration of contact (Dynamic test, T = 40 °C).

According to the results observed in Fig. 6, in the experiments on the solubility of asphaltene, increasing the duration of contact from 30 to 60 min does not affect the solubility of asphaltene. Conversely, in the experiments on the solubility of asphaltic sludge, increasing the duration of contact from 30 to 60 min has a direct impact on the solubility of asphaltic sludge; as the extended contact time allows for greater penetration of solvent ions into the asphaltic sludge, resulting in a 10% increase in solubility.

**Fig. 7:**
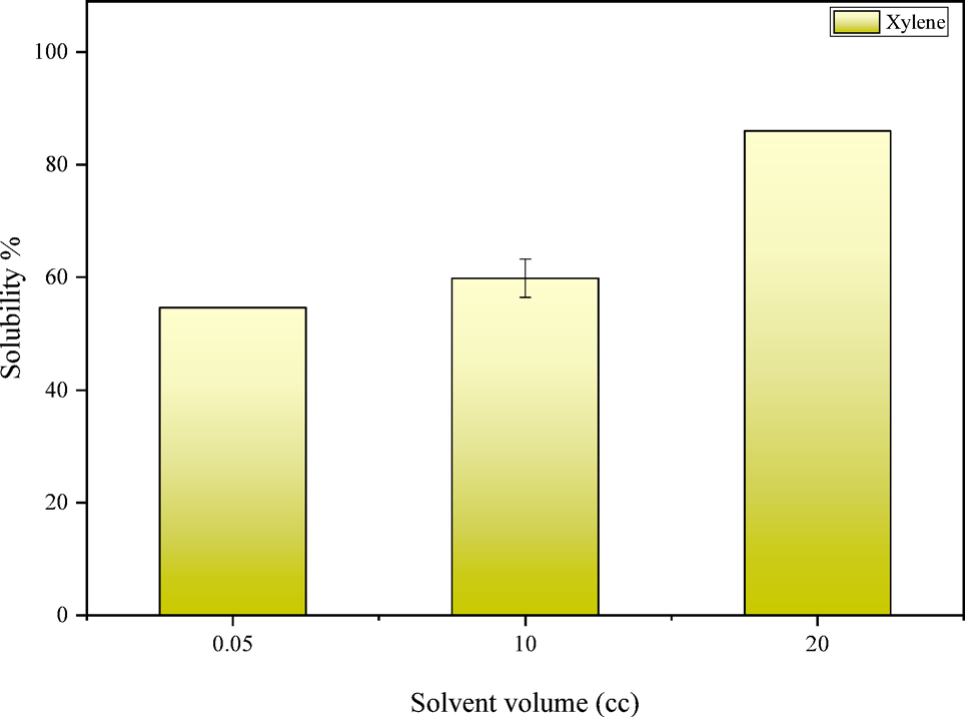
Results of the effect of solvent volume (Dynamic test, T = 40 °C).

As shown in Fig. 7, with an increase in the amount of solvent, the percentage of solubility increases, and this relationship is direct. As the amount of solvent increases, the number of reacting ions also increases (the quantity of reactant material rises), resulting in a greater amount of asphaltic sludge being dissolved. The increase in solubility in these experiments shows an upward trend, with each increment in solvent amount adding 15 to 20 percent to the solubility.

## Perspectives and limitations

This study focused on evaluating the performance of different solvents in removing asphaltic sludge formed during acidizing operations. While the findings provide insights into solvent-based removal methods, several limitations should be considered, and potential directions for future research are proposed below:

- The experiments were carried out at atmospheric pressure and a moderate temperature of 40 °C. However, in real reservoir and wellbore conditions, temperatures and pressures can be significantly higher. To better simulate field conditions, it is recommended that future studies be conducted under high-temperature and high-pressure (HTHP) conditions.
- All experiments in this study were performed in open vessels. In reality, sludge removal occurs within porous media in the reservoir. Therefore, it is advisable to perform similar tests using core samples and core flooding setups to evaluate solvent penetration and sludge displacement under dynamic flow conditions.
- The asphaltic sludge samples used in this work were in dry form. Conducting experiments on the undried form would allow better evaluation of the effect of solvent behavior under actual field-like conditions.

## Conclusions

In this study, the solubility of asphaltic sludge was specifically examined using four different solvents, and the effects of parameters such as the presence of powdered stone, duration of contact, amount of solvent, and stirring speed were tested. The following results were obtained:

1. Tetrahydrofuran and xylene were identified as two effective solvents for the solubility of asphaltic sludge, with the solubility of tetrahydrofuran under laboratory conditions being 70%, while the solubility of xylene under laboratory conditions is 60%.
2. The presence of powdered stone has an inverse relationship with solubility, meaning that with the presence and increase of powdered stone in the solution, solubility decreases. According to the conducted experiments, the solubility drops to less than half of the level observed in the absence of powdered stone.
3. The duration of contact between the solvent and solute has a direct relationship with solubility, such that with an increase in the duration of contact, solubility increases by 10 percent.
4. The amount of solvent has a direct relationship with solubility, meaning that as the quantity of solvent increases, solubility increases by 10 to 20 percent. This increase in solubility is dependent on the amount of solvent used.
5. The stirring speed has a direct relationship with solubility, meaning that as the stirring speed increases, solubility also increases. Based on the examined stirring speeds, solubility increases by 15 percent.
6. The solubility of asphaltic sludge is dependent on the type of components and elements present in the sludge. Factors such as the presence of iron ions in the sludge, the pH level, and the amount of asphaltene.

## Data Availability

All data generated or analyzed during this study are included in this manuscript.
